# Human papillomavirus 16 (HPV 16) E6 but not E7 inhibits the antitumor activity of LKB1 in lung cancer cells by downregulating the expression of KIF7

**DOI:** 10.1111/1759-7714.13640

**Published:** 2020-09-18

**Authors:** Yue Hu, Ming‐Zhe Wu, Na‐Jin Gu, Hong‐Tao Xu, Qing‐Chang Li, Guang‐Ping Wu

**Affiliations:** ^1^ Department of Pathology, The First Affiliated Hospital and College of Basic Medical Sciences China Medical University Shenyang China; ^2^ Department of Pathology First Hospital of Qinhuangdao Qinhuangdao China; ^3^ Departments of Gynecology The First Hospital of China Medical University Shenyang China

**Keywords:** Human papillomavirus (HPV), kinesin family member 7 (KIF7), liver kinase B1 (LKB1), lung cancer, serine/threonine kinase 11 (STK11)

## Abstract

**Background:**

The E6 and E7 proteins in human papillomavirus 16 (HPV 16) are the main oncogenes in the occurrence of lung cancer. In recent studies, we found that E6 and E7 downregulated the expression of LKB1 in lung cancer cells. However, it is still unclear how E6 and E7 regulate LKB1 in lung cancer cells.

**Methods:**

Double directional genetic manipulation and nuclear plasma separation technology were performed to explore the molecular mechanism of E6 and E7 inhibiting the antitumor activity of LKB1 in well‐established lung cancer cell lines.

**Results:**

E6 but not E7 significantly downregulated the expression of tumor suppressor KIF7 at protein level, and the inhibition of KIF7 further reduced the expression of LKB1 both in the nuclei and in the cytoplasm, whereas reduced the expression of p‐LKB1 in the cytoplasm only. This suggested that HPV 16 E6 but not E7 downregulates the antitumor activity of LKB1 by downregulating the expression of p‐LKB1 in the cytoplasm only.

**Conclusions:**

Here, we demonstrated for the first time that E6 but not E7 inhibits the antitumor activity of LKB1 in lung cancer cells by downregulating the expression of KIF7. Our findings provide new evidence to support the important role of KIF7 in the pathogenesis of lung cancer and suggests new therapeutic targets.

## Graphical abstract

We demonstrated for the first time that E6 but not E7 inhibits the antitumor activity of LKB1 in lung cancer cells by downregulating the expression of KIF7 and the antitumor activity of LKB1 was determined by two factors, one was phosphorylation of LKB1, the other was that p‐LKB1 must be located in the cytoplasm, both were indispensable.

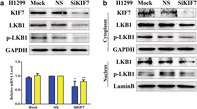

## Introduction

In 1980, Syrjänen first proposed the hypothesis of the role of HPV infection in the occurrence of bronchosquamous cell carcinoma.^1^ With the rapid development of molecular biology technology and its timely application in the study of lung cancer, researchers found that E6 and E7 proteins in human papillomavirus 16 (HPV 16) were the main oncogenes, with long‐term persistent infection often related to the occurrence of lung cancer, especially in non‐smokers and Asians.^2^, ^3^, ^4^, ^5^ The E6 protein has been previously reported to inhibit cell apoptosis mainly by degrading the p53 gene,^6^ while the E7 protein has been found to promote cell proliferation mainly by inhibiting the retinoblastoma protein (pRb).^7^ Therefore, the signaling pathways regulated by E6 and E7 proteins might not be exactly the same in the occurrence of lung cancer. Recently, we found that the overexpression of both E6 and E7 in HPV 16 downregulated the expression of LKB1 at both protein and mRNA levels in lung cancer cells.^8^, ^9^ However, the potential molecular mechanism of regulation of LKB1 by E6 and E7 is not clear.

Liver kinase B1 (LKB1), also known as serine/threonine kinase 11 (STK11), was first found to be a tumor suppressor gene in patients with Peutz‐Jeghers syndrome (PJs).^10^, ^11^ It has serine/threonine protein kinase activity and plays an important role in the occurrence and development of lung cancer by phosphorylating substrate protein or binding with target protein to regulate gene expression.^12^, ^13^ It has been previously shown that LKB1 is a critical barrier to lung tumorigenesis, controlling the occurrence, differentiation and metastasis of lung tumorigenesis.^14^ In a study by Tsai *et al*. the in vitro experiment results showed that the phosphorylation of LKB1 at Ser 428 was involved in the anticancer effect of human oral cancer cells.^15^ Recently, Wong *et al*. found that kinesin family member 7 (KIF7) promoted the antitumor activity of LKB1 through upregulation of the expression of LKB1 and induction of phosphorylation of LKB1 at Ser 428.^16^

KIF7 is on 15q26.1 and is a member of the kinesin‐4 family that has been shown to play critical roles in primary cilia formation and Hedgehog (Hh) signaling in embryonic development.^17^, ^18^ KIF7 has been reported to be a novel tumor suppressor in prostate cancer that acts by suppressing proliferation, migration, invasion and tumorigenesis through the LKB1/PTEN/AKT signaling pathway.^16^ Low expression of KIF7 has been reported to indicate poor prognosis in epithelial ovarian cancer^19^ and facilitate cell survival and migration of choriocarcinoma cells.^20^

In this study, we investigated whether HPV16 E6 downregulated the expression of p‐LKB1 through the HPV‐KIF7‐LKB1 axis, and report that HPV16‐E6 but not E7 inhibited the antitumor activity of LKB1 in lung cancer cells by downregulating the expression of KIF7.

## Methods

### Cell culture

Three human non‐small cell lung carcinomas (NSCLC) cell lines (H460, H1299, A549), and normal human bronchial epithelial (HBE) cell line were used in this study. HBE, H460, H1299, and A549 cell lines were obtained from the ATCC (Manassas, VA, USA) and cultured in RPMI‐1640 medium, supplemented with 10% fetal bovine serum (FBS, Cellmax, Beijing, China) at 37°C in a 5% CO_2_ humidified atmosphere.

#### Transfection and interference

Based on the previous study results, H1299 cells were E6 and E7 low‐expression cell lines, while A549 cells were E6 and E7 high‐expression cell lines.^9^ The pEGFP‐N1‐HPV16 E6, pEGFP‐N1‐HPV16 E7, and pEGFP‐N1 plasmids were kindly provided by Prof Xudong Tang, Institute of Biochemistry and Molecular Biology, Guangdong Medical College, China. HPV16 E6 SiRNA and HPV16 E7 SiRNA were purchased from RIBOBIO (Guangzhou, China), KIF7 siRNA was purchased from General Biosystems (Anhui, China). Disordered siRNA was used as a nonspecific siRNA control.

The cells were transfected into six‐well plates using Lipofectamine 3000 (Invitrogen, Carlsbad, CA, USA) according to the manufacturer's instructions. Transfection with empty vector and mock transfection were used as controls. The protein analysis was assessed 48 hours after transfection by western blotting. The mRNA analysis was assessed 24 hours after transfection by quantitative real‐time reverse transcriptase‐polymerase chain reaction (qRT‐PCR).

### Western blot assays

The assays were performed as previously described.^9^ Information about primary antibodies is as follows: HPV16 E6 (1:100, Bioss Biotechnology Co., Ltd., Beijing, China), HPV16 E7 (1:100, Bioss Biotechnology Co., Ltd., Beijing, China), KIF7 (1:1000, Proteintech, Wuhan, China), LKB1 (1:800, Proteintech, Wuhan, China), p‐LKB1 (Phospho‐Ser428) (1:800, Sangon Biotech, Shanghai, China), GAPDH (1:1000, Cell Signaling Technology, Danvers, MA, USA), and Lamin B (1:800, WanLeibio, Shenyang, China).

### Quantitative real‐time PCR

Total RNA was extracted from cells with Trizol solution (TaKaRa, Dalian, China) and RNase RNA isolation kit (QIAGEN, Hilden, Germany) according to the manufacturer's instructions. A total of 1 μg RNA was subjected to reverse transcription reaction to obtain cDNAs by using a Prime Script RT reagent Kit (TaKaRa, Dalian, China). qRT‐PCR was performed using SYBR Premix Ex Taq II (TaKaRa, Dalian, China) on 7900HT Fast Real‐Time PCR System (Applied Biosystems). Nontemplate controls were carried out each time for each primer pair to detect nonspecific amplification. Glyceraldehyde‐3‐phosphate dehydrogenase (GAPDH) was amplified as the internal control. All reactions were run in triplicate. The fold change of mRNA expression was calculated using the 2^−ΔΔct^ method. Detailed information of the primers is given in Table 1.

**Table 1:** Sequences and features of primers used for qRT‐PCR

Gene	Forward/reverse	Sequence	Size (bp)	mRNA
E6	270	GTATGGAACAACATTAGAACAGCAA	79	KX545363
	349	GTGGCTTTTGACAGTTAATACACC		
E7	482	GCATGGAGATACACCTACATTG	273	KX545363
	754	TGGTTTCTGAGAACAGATGG		
LKB1	223	AGGGCCGTCAAGATCCTCAA	187	KU178339
	409	GCATGCCACACACGCAGTA		
KIF7	2496	GGAGAAGAAGCAGGCTACGG	374	NM_198525.3
	2869	GCTGCTGTAGCACCTTCTCC		
GAPDH	50 120	TTCTTTTGCGTCGCCAGCCGAG CCAGGCGCCCAATACGACCAAA	71	XM_019023188.1


mRNA, messenger RNA; qRT‐PCR, quantitative real‐time reverse transcriptase‐polymerase chain reaction.

### Nuclear plasma separation technology

KIF7‐specific siRNA was applied to knockdown the expression of KIF7 in the H1299 cell line. Nuclear and cytoplasmic protein extraction kit (P0028, Beyotime, Shanghai, China) was used to perform nuclei isolation. All the experiments were performed on ice, cytoplasmic protein extraction reagent A with 1 mM PMSF was added to the cell suspension, followed by 10–15 minutes of incubation on ice after five seconds of vigorous oscillation at the highest speed. Cytoplasmic protein was then added to the cytoplasmic protein extraction reagent B. After five seconds of vigorous oscillation at the highest speed, the supernatant was centrifuged at 4°C at 12 000 *g* for five minutes. The nuclear protein extraction reagent with 1 mM PMSF was then added to the remaining precipitate, which was vortexed vigorously at the highest speed for 15–30 seconds and then placed in an ice bath for 30 minutes, during which time the nuclear protein was intensely vortexed for 15–30 seconds every 1–2 minutes. After centrifugation at 4°C and 12 000 *g* for 10 minutes, the supernatant was absorbed into a precooled plastic tube, and the nucleoprotein was located in the supernatant.

### Statistical analysis

SPSS 22.0 software was utilized for statistical analyses in this study. Each assay was performed at least three times. The data were expressed as mean ± SD. Statistical significance was determined by Student's *t*‐test, and a *P*‐value <0.05 was considered statistically significant.

## Results

### Screening of lung cancer cell lines

Based on our previous results, low expression of E6 and E7 was found in the H1299 cell line, and high expression of E6 and E7 was found in the A549 cell line.^21^ The expression of KIF7 was screened in the normal bronchial epithelial cell line HBE and lung cancer lines H1299, H460, and A549, respectively. As shown in Fig S1, a high expression level of KIF7 was found in HBE and H1299, whereas low expression level of KIF7 was observed in H460 and A549 cell lines. Further assays were designed and performed based on these results.

The overexpression of E6 significantly downregulated the expression of KIF7 and LKB1, whereas the overexpression of E7 significantly downregulated the expression of LKB1 only.

**Figure 1:**
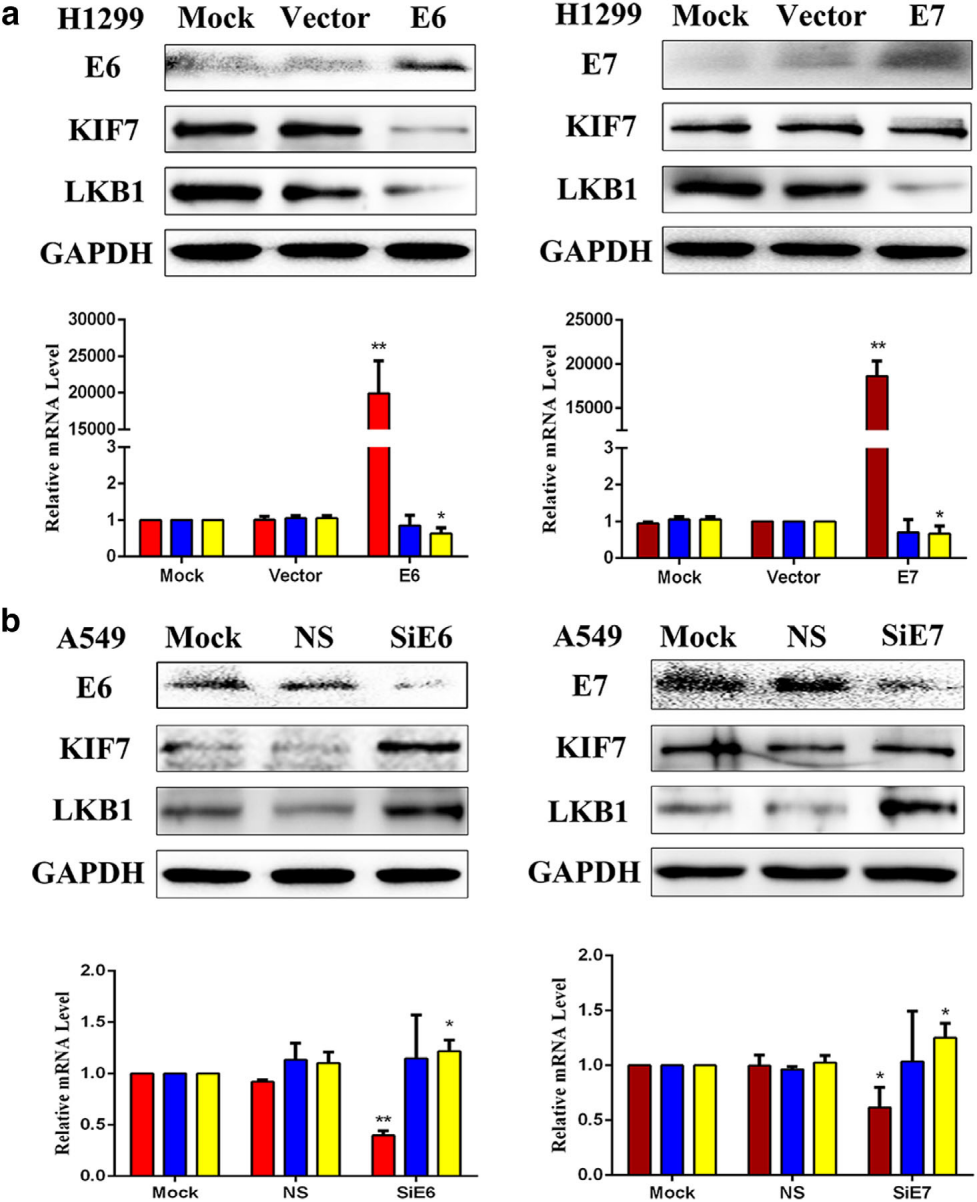
(**a**) The regulation of E6 and E7 on the expression of KIF7 and LKB1 by transfection. 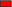, E6; 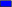, KIF7; 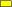, LKB1; 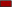, E7; 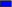, KIF7; 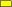, LKB1; and (**b**) interference 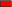, E6; 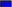, KIF7; 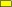, LKB1; 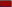, E7; 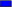, KIF7; 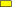, LKB1, in lung cancer cells. The expression levels of E6, E7, KIF7, and LKB1 were demonstrated by western blotting and RT‐qPCR in lung cancer cells. Mock, mock transfection; NS, no significance; Vector, empty vector (*P < 0.05; **P < 0.01).

The pEGFP‐N1‐E6 or E7 vectors were transiently transfected into the low expression H1299 cell line, and the E6 or E7 empty vectors and mock transfections served as controls. The results showed that the overexpression of E6 significantly downregulated the expression of KIF7 at protein level only and the expression of LKB1 at both protein and mRNA levels, whereas the overexpression of E7 significantly downregulated the expression of LKB1 at both protein and mRNA levels and had no regulatory effect on KIF7. The results are shown in Fig 1a.

The inhibition of E6 evidently upregulated the expression of KIF7 and LKB1, whereas the inhibition of E7 evidently upregulated the expression of LKB1 only.

To further verify the regulatory roles of E6 or E7 on KIF7 and LKB1, we applied E6 or E7‐specific siRNA to knockdown the expression of E6 or E7 in the A549 cell line. E6 or E7‐nonspecific siRNA and mock specific siRNA served as the controls. The results indicated that the inhibition of E6 evidently upregulated the expression of KIF7 at protein level only and the expression of LKB1 at both protein and mRNA levels, whereas the inhibition of E7 evidently upregulated the expression of LKB1 at both protein and mRNA levels and had no regulatory effect on KIF7. The results are presented in Fig 1b.

The inhibition of KIF7 obviously downregulated the expression levels of LKB1 and p‐LKB1.

**Figure 2:**
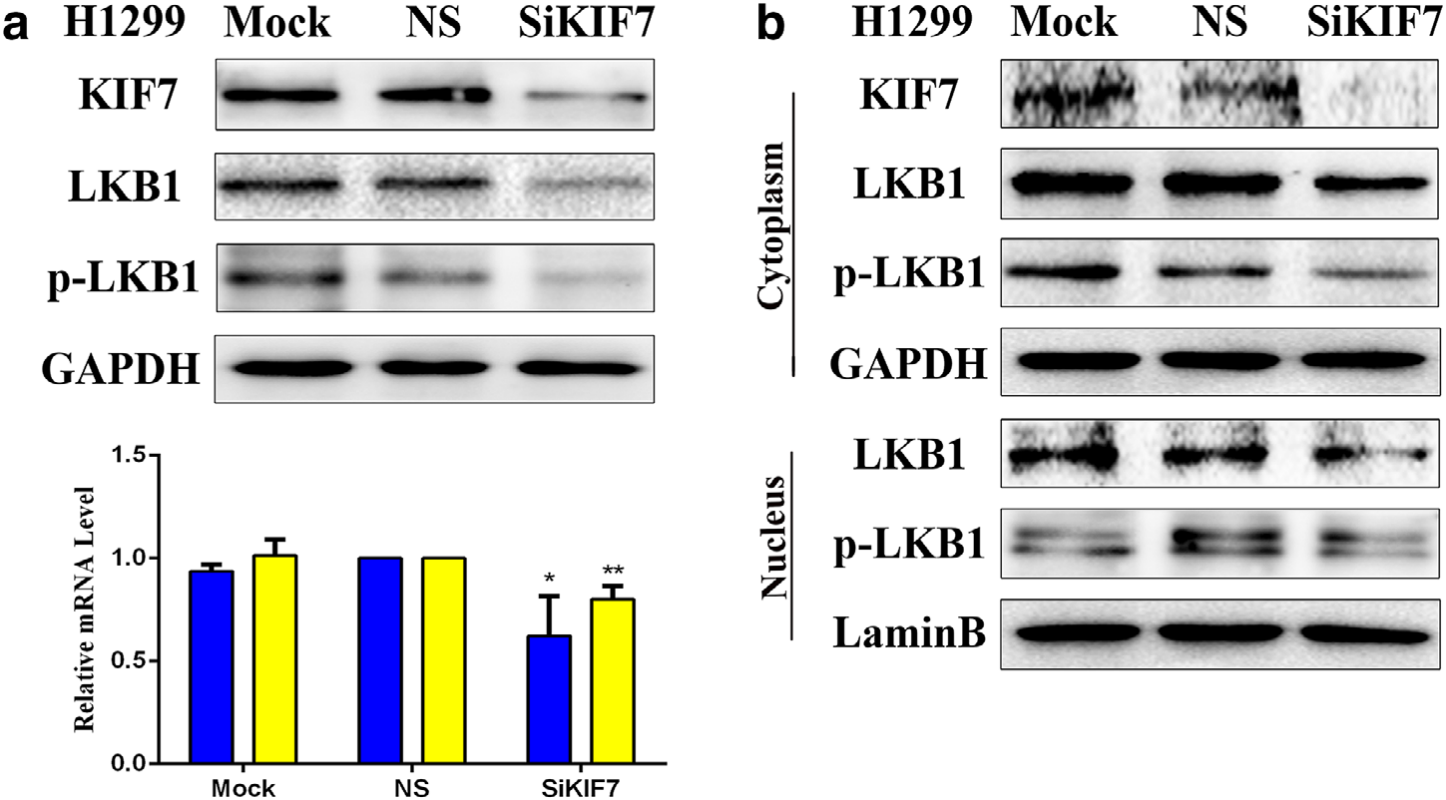
(**a**) The regulation of KIF7 on the expression of LKB1 and p‐LKB1 by interference, and the expression levels of KIF7, LKB1 and p‐LKB1 were demonstrated by western blotting in H1299 cells. 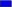, KIF7; 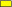, LKB1. (**b**) The inhibition of KIF7 downregulated the expression levels of LKB1 in both the cytoplasm and nuclei, whereas the expression level of p‐LKB1 was only downregulated in the cytoplasm. KIF7‐specific siRNA was performed by western blotting in H1299 cells. Mock, mock transfection; NS, no significance (*P < 0.05; **P < 0.01).

To further verify the regulatory roles of KIF7 on LKB1 and p‐LKB1, we applied KIF7‐specific siRNA to knockdown the expression of KIF7 in the H1299 cell line. KIF7‐nonspecific siRNA and mock specific siRNA served as the controls. The results indicated that the inhibition of KIF7 obviously downregulated the expression of LKB1 at both protein and mRNA levels and the expression of p‐LKB1 at protein level. The results are presented in Fig 2a.

The loss of KIF7 reduced the expression of LKB1 both in the nuclei and cytoplasm, whereas reduced the expression of p‐LKB1 in the cytoplasm only.

To determine whether localization of LKB1 or p‐LKB1 was related to the regulation of KIF7, we used western blotting to detect the expression of LKB1 or p‐LKB1 in nucleic and cytoplasmic fractions. We knocked down KIF7 in H1299 cells and separated the proteins in the nucleus and in the cytoplasm by using nuclear plasma separation technology. We found that the expression level of LKB1 was downregulated in both the nuclei and cytoplasm, whereas the expression level of p‐LKB1 was downregulated in the cytoplasm only. The results are presented in Fig 2b.

## Discussion

In our previous studies, we found that the overexpression of both E6 and E7 in HPV 16 downregulated the expression of LKB1 at both protein and mRNA levels in lung cancer cells.^8^, ^9^ However, the underlying molecular mechanism of the regulation of LKB1 by HPV16 is unclear. In this study, we found that the overexpression of E6 significantly downregulated the expression of KIF7 at protein level only and the expression of LKB1 at both protein and mRNA levels. Conversely, the knockdown of E6 evidently upregulated the expression of KIF7 at protein levels only and the expression of LKB1 at both protein and mRNA levels. We speculated whether the regulation mechanism of E6 on KIF7 might be through translational or post‐translational pathways. However, we found that whether by transfection or interference, E7 had no regulatory effect on KIF7. Although E7 could also downregulate the expression of LKB1, it was not achieved by downregulating KIF7. Therefore, we speculated that the regulation of LKB1 by E7 might be accomplished by other genes. To our knowledge, this is the first time that KIF7 has been found to be involved in HPV16 E6 but not E7 by downregulating the expression of LKB1 in lung cancer cells. The detailed regulatory mechanism between E6 and KIF7 needs to be studied further in the future.

The increased expression of protein and mRNA of LKB1 does not mean that its antitumor activity is increased. Wong *et al*. speculated that KIF7 upregulated the antitumor activity of LKB1 in two ways; one was phosphorylation, and the other subcellular localization. They also found that KIF7 significantly upregulated the expression of LKB1 in both the nuclei and cytoplasm and promoted the phosphorylation of LKB1 at Ser 428.^16^ However, they did not find that the expression level of LKB1 phosphorylation induced by KIF7 in the nucleus was completely different from that in the cytoplasm. In the present study, we found that inhibition of KIF7 not only significantly downregulated the expression of LKB1, but also downregulated the expression of p‐LKB1. Our results from nuclear‐cytoplasmic protein separation western blot analysis showed that the inhibition of KIF7 significantly decreased the expression level of p‐LKB1 at Ser 428 in the cytoplasm, but the expression level in the nucleus was basically unchanged. These results indicate that the antitumor activity of LKB1 is determined by two factors; phosphorylation of LKB1, and that p‐LKB1 must be located in the cytoplasm; both were indispensable. In other words, the activation state of LKB1 was that the expression of p‐LKB1 in cytoplasm was significantly increased. It is not clear which of the two conditions occurs first; the phosphorylation or subcellular translocation. The detailed molecular mechanism needs further study in the future.

We previously carried out a series of studies on the role of HPV16 in the carcinogenesis of lung cancer, and found that the carcinogenic effects of E6 and E7 proteins were completely consistent with other studies.^8^, ^9^, ^21^ However, Liu *et al*. determined that E6 and E7 in HPV16 played different roles in the regulation of ERK signaling pathway.^22^ For the first time in this study, we found that HPV16‐E6 but not E7 inhibited the antitumor activity of LKB1 in lung cancer cells by downregulating the expression of KIF7.

In conclusion, here we demonstrated for the first time that E6 but not E7 inhibits the antitumor activity of LKB1 in lung cancer cells by downregulating the expression of KIF7 and that the antitumor activity of LKB1 is determined by two factors; the phosphorylation of LKB1, and that p‐LKB1 must be located in the cytoplasm, both were indispensable. Our findings provide new evidence to support the important role of KIF7 in the pathogenesis of lung cancer and suggest new therapeutic targets.

## Disclosure

The author(s) declare that there are no potential conflicts of interest with respect to the research, authorship, and/or publication of this article.

## Supporting information


**Figure S1** Detection of the expression of KIF7 was in lung cancer cell lines (H1299, H460, and A549) using western blotting; HBE, a normal bronchial epithelial cell line, served as the positive control, and GAPDH served as the internal control.
