# Supplementary figures and images for: Human papillomavirus 16 (HPV 16) E6 but not E7 inhibits the antitumor activity of LKB1 in lung cancer cells by downregulating the expression of KIF7
Source: Thorac Cancer. 2020 Sep 18;11(11):3175–80. doi: 10.1111/1759-7714.13640 (PMC7606012; doi:10.1111/1759-7714.13640)

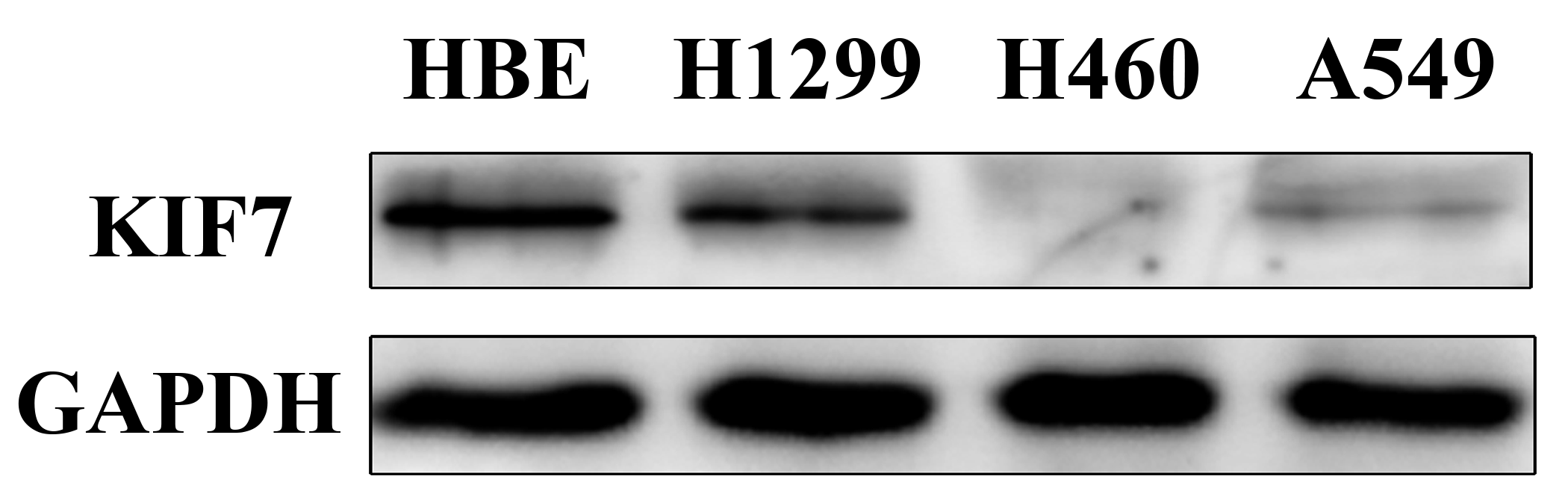

Supplement: Supplementary file 1 — Figure S1 Detection of the expression of KIF7 was in lung cancer cell lines (H1299, H460, and A549) using western blotting; HBE, a normal bronchial epithelial cell line, served as the positive control, and GAPDH served as the internal control. [file TCA-11-3175-s001.tif]
